# Synaptonemal Complex Components Persist at Centromeres and Are Required for Homologous Centromere Pairing in Mouse Spermatocytes

**DOI:** 10.1371/journal.pgen.1002701

**Published:** 2012-06-28

**Authors:** C. Gaston Bisig, Michel F. Guiraldelli, Anna Kouznetsova, Harry Scherthan, Christer Höög, Dean S. Dawson, Roberto J. Pezza

**Affiliations:** 1 Ciquibic-Conicet, Universidad Nacional de Cordoba, Cordoba, Argentina; 2 Oklahoma Medical Research Foundation, Oklahoma City, Oklahoma, United States of America; 3 Department of Cell and Molecular Biology, Karolinska Institutet, Stockholm, Sweden; 4 Institut für Radiobiologie der Bundeswehr, Universität Ulm, Munich, Germany; 5 Department of Cell Biology, Oklahoma University Health Science Center, Oklahoma City, Oklahoma, United States of America; Stowers Institute for Medical Research, United States of America

## Abstract

Recent studies in simple model organisms have shown that centromere pairing is important for ensuring high-fidelity meiotic chromosome segregation. However, this process and the mechanisms regulating it in higher eukaryotes are unknown. Here we present the first detailed study of meiotic centromere pairing in mouse spermatogenesis and link it with key events of the G2/metaphase I transition. In mouse we observed no evidence of the persistent coupling of centromeres that has been observed in several model organisms. We do however find that telomeres associate in non-homologous pairs or small groups in B type spermatogonia and pre-leptotene spermatocytes, and this association is disrupted by deletion of the synaptonemal complex component SYCP3. Intriguingly, we found that, in mid prophase, chromosome synapsis is not initiated at centromeres, and centromeric regions are the last to pair in the zygotene-pachytene transition. In late prophase, we first identified the proteins that reside at paired centromeres. We found that components of the central and lateral element and transverse filaments of the synaptonemal complex are retained at paired centromeres after disassembly of the synaptonemal complex along diplotene chromosome arms. The absence of SYCP1 prevents centromere pairing in knockout mouse spermatocytes. The localization dynamics of SYCP1 and SYCP3 suggest that they play different roles in promoting homologous centromere pairing. SYCP1 remains only at paired centromeres coincident with the time at which some kinetochore proteins begin loading at centromeres, consistent with a role in assembly of meiosis-specific kinetochores. After removal of SYCP1 from centromeres, SYCP3 then accumulates at paired centromeres where it may promote bi-orientation of homologous centromeres. We propose that, in addition to their roles as synaptonemal complex components, SYCP1 and SYCP3 act at the centromeres to promote the establishment and/or maintenance of centromere pairing and, by doing so, improve the segregation fidelity of mammalian meiotic chromosomes.

## Author Summary

Meiosis is the key developmental program of gametogenesis during which haploid gametes are generated to cope with the doubling chromosome number that occurs after fertilization. During the first meiotic division, homologous chromosomes pair, recombine, and dissociate. Errors in any of these processes are commonly linked to human infertility, spontaneous abortions, and severe aneuploidy-based birth defects. Chiasmata, the cytological manifestations of recombination, provide a physical link that holds the homologous maternal and paternal chromosomes in pairs, facilitating their segregation, and that guarantees each gamete receives only one copy of each chromosome. Recently, however, studies in lower eukaryotes have suggested that, in the absence of recombination, pairing between centromeric regions promotes proper segregation of these non-recombined meiotic homologous chromosomes and contributes significantly to the segregation fidelity of chromosomes that have recombined. We show here that the SYCP1 and SYCP3 proteins are required for centromere pairing in mouse. Our findings define structures and possible mechanisms of a process that was not previously known to occur in mammals, which may act to insure that the correct numbers of chromosomes are transferred to the next generation.

## Introduction

During the first meiotic division, homologous chromosomes pair, recombine and dissociate. Successful completion of these processes is required for a pair of homologous chromosomes (bivalent) to mount the meiotic spindle. Organization of the chromosomes into pairs ensures orderly segregation of homologous chromosomes to opposite spindle poles at the first meiotic division, ensuring that each gamete receives one copy of each chromosome. Errors in meiotic homologous chromosome segregation are the leading cause of human aneuploidy. Some examples of hereditary diseases caused by aneuploidies are several types of Ataxias and Down, Klinefelter, Edwards and Turner Syndromes [1]. The molecular basis of aneuploidy in humans is poorly understood. Identification and description of mechanisms used to promote meiotic fidelity are essential to improve this understanding.

One component of the meiotic process that is critical for ensuring high fidelity chromosome segregation is recombination. Chiasmata, the cytological manifestation of product of recombination, provide a physical link that holds the homologs in pairs and facilitates their orientation on the spindle at meiosis I [2]. Consequently, mutations that reduce the amount of recombination are invariably associated with increased errors in meiotic chromosome segregation. Indeed, in yeast, mice, and humans, chromosome pairs that fail to recombine have an increased chance of mis-segregating [3]. However, in yeast and flies it has been shown that such error-prone chromosomes do not segregate randomly. Instead, a high proportion of them are partitioned correctly using segregation-promoting mechanisms that lack chiasmata [4]. In yeast, pairing between centromeres promotes proper segregation of non-recombined meiotic chromosomes [5] and also contributes significantly to the segregation fidelity of chromosomes that have recombined [6]. Similarly, in Drosophila, pairing between blocks of peri-centric heterochromatin has been shown to orient non-recombined partner chromosomes that have not become tethered by a chiasmata [7], [8]. We tested the hypothesis that in a mammalian system, centromere pairing links homologous centromeres in a way that has been shown in other model systems to promote their proper attachment to microtubules from opposite poles of the meiotic I spindle. While centromere pairing has been observed in a number of organisms (reviewed in [2]), its existence and roles in meiosis of mammals have not been explored.

An intriguing characteristic of centromere pairing is that synaptonemal complex components in yeast (Zip1) and Drosophila (C(3)G) are required for maintenance of centromere pairing late in meiotic prophase [6], [9], [10]. The mammalian synaptonemal complex protein SYCP1 appears to be the functional homolog of Zip1 and C(3)G [11]. Previous work has shown that *Sycp1* knockout spermatocytes reach advanced stages of prophase I with an absence of homolog synapsis including the centromeres [12]. In this work we explore whether SYCP1 and SYCP3 are present at the paired centromeres in earlier and later stages of mouse spermatogenesis and discuss the possible significance of centromere pairing with closely linked processes such as kinetochore assembly or bi-orientation of centromeres at the meiosis I division.

A possible function of centromere pairing is to promote the proper loading/assembly of kinetochore-specific proteins at the meiotic kinetochore. Kinetochores are located on the outside face of centromeres and function to attach chromosomes to the meiosis I spindle. Ultrastructurally, kinetochores are distinct trilaminar structures. On mitotic chromosomes, there is an inner plate, constituted by chromatin containing a centromeric histone H3 variant, CENPA, auxiliary proteins and DNA. There is an outer plate composed of proteins involved in the process of binding microtubules. The region between the two mitotic sister kinetochores is called the inner centromeric domain and is defined by the presence of a chromosome passenger complex (INCENP, AURORA B, SURVIVIN, BOREALIN and AURORA C) (reviewed in [13]). Assembly of mitotic kinetochore structures on meiotic chromosomes could lead to segregation of sister chromatids away from each another at meiosis I, which is thought to be one of the most common types of meiotic segregation errors in humans. Little is known about the process by which cells assemble meiotic rather than mitotic centromere structures. However, recent studies in mouse spermatocytes have made clear there is a program of assembly of the meiosis I outer kinetochore after the time at which chromosomes have synapsed with their partners in prophase I and when sister kinetochores are tightly cohered [14]. Persistence of synaptonemal complex components at the centromeres as the cell progresses towards metaphase has been demonstrated in model organisms [6], [9], [10]. This may suggest new roles for these synaptonemal complex proteins at the centromere. Indeed we find that SYCP1 and SYCP3 proteins remain at centromeres co-incident with the time of outer kinetochore assembly, and appear to promote steps in the establishment and/or maintenance of centromere pairing until the centromeres begin their attachment to the meiotic spindle.

## Results

### Centromere versus telomere associations in early mouse prophase

We have characterized events of centromere pairing in male mouse meiosis by analyzing the kinetics of centromere pairing in cells at different stages of prophase in meiosis I. Parallel observations are made by Qiao et al. in the accompanying study [15].

**Figure 1:**
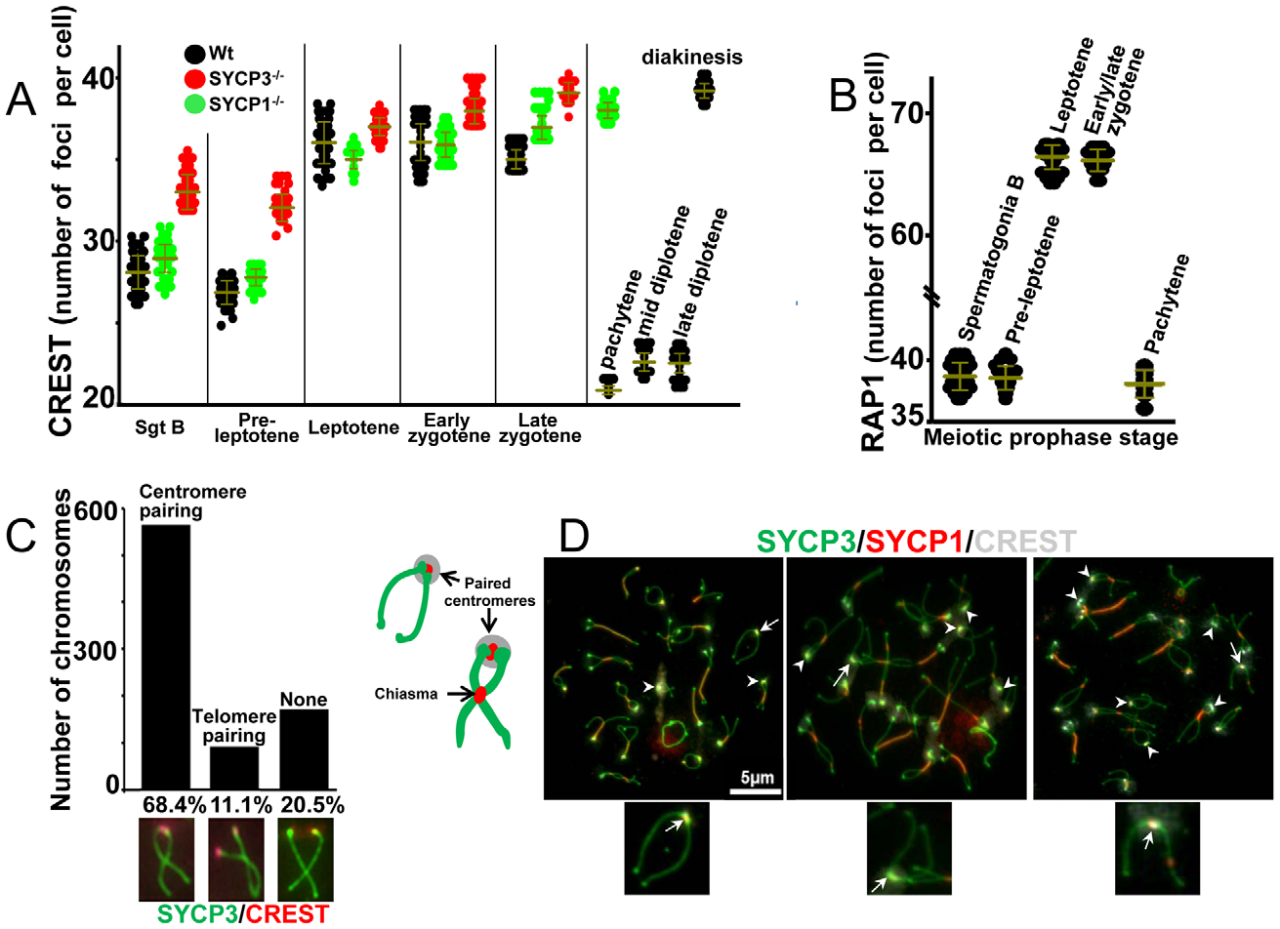
Dynamics of centromere pairing in mouse spermatocytes. (A) Dynamics of centromere pairing observed in squashed spermatocytes of wild-type mice at meiotic prophase I. In wild-type spermatocytes the number of CREST foci remained constant as cells progressed from pachytene to diplotene (22.9±0.7 and 22.7±0.9 CREST foci for mid and late diplotene respectively) consistent with centromere pairing. Squashed spermatocytes from *Sycp3* knockout mice exhibited increased numbers of CREST foci in very early prophase and *Scyp1* knockout mice exhibited elevated CREST foci at pachytene consistent with an absence of centromere pairing. Horizontal lines denote the means and vertical lines denote standard deviations. See Table 1 for summary of means, standard deviation and results of statistical tests. (B) The number of RAP1 signals was used to determine the extent of telomeres association in squashed spermatocytes of wild-type mice at meiotic prophase I. Horizontal lines denote the means and vertical lines denote standard deviations. See Table 1 for summary of means and standard deviation. (C) Quantification of centromere pairing was carried out by scoring the number of homologous chromosomes in spermatocytes spreads at the diplotene stage experiencing central chiasmata, and with paired centromeres but unpaired distal telomeres, paired distal telomeres but unpaired centromeres or absence of both centromere and telomere pairing. Note that centromeres are preferentially paired over telomeres. (D) Centromere pairing was analyzed in surface chromosome spreads of wild-type spermatocytes. The cartoon represents homologous chromosomes at mid-diplotene tethered by chiasmata and paired centromeres. Arrowheads indicate paired centromeres for chromosomes undergoing one exchange. Tailed white arrows indicate the single physical link connecting apparent non-exchange homologous chromosomes. Magnification bar represents 5 µm and correspond to all images but only show in one panel.

To score the number of centromeres we immunostained structurally intact preserved nuclei from squashed seminiferous tubules with CREST antibodies specific for kinetochores (Figure 1 and Figure S1). Mice have 20 pairs of chromosomes (in males 19 pairs of somatic chromosomes and a pair of heteromorphic sex chromosomes). The sex chromosomes pair only at the PAR region, which includes the distal telomeric but not the centromeric region. Complete pairing of homologous centromeres in pachytene spermatocytes would be expected to yield 21 centromeric CREST foci while completely dispersed centromeres would yield 40 foci.

We first analyzed cells in the very early stages of spermatogenesis. B type spermatogonia, which are engaged in pre-meiotic proliferation, show an average of 28.5±1.9 CREST foci per cell (n = 100). For pre-leptotene spermatocytes, which are in meiotic S-phase, or have just passed premeiotic S, as shown by incorporation of EDU into replicating DNA (Figure S1), we observed an average of 27.1±1.8 CREST foci per cell (n = 100) (Figure 1A and Table 1). This intermediate level of CREST foci could result from either partial pairing of centromeres, or from telomere pairing; because each centromere is adjacent to one of the telomeres of the chromosome, one-half of randomly paired telomeres would bring together two centromeres and reduce the number of CREST foci from 40 to about 30. To distinguish between these possibilities we stained chromosomes for the telomere-associated protein, RAP1 [16] (Figure 1B and Table 1). Most nuclei in early prophase exhibited almost exactly the number of foci predicted if all of the telomeres are in pairwise associations (39.2±1.8 and 39.1±1.6 RAP1 foci for B type spermatogonia and pre-leptotene cells respectively). These results suggest that most of the 80 telomeres are arranged in pairs or clusters in early prophase nuclei. To test whether this pairing was between homologous regions, we performed fluorescence in-situ hybridization (FISH), with a point probe specific for the centromeric region of chromosome VIII (which is associated with the proximal telomere). The results showed that in most B type spermatogonia and pre-leptotene meiocytes this centromere and its nearby telomere are not associated with their homologous partners (only 17% homologous pairing was observed for early prophase cells. Figure S2). Together these results suggest that in very early meiosis in mouse spermatocytes, centromeres do not exhibit an extended period of centromere coupling as seen in some other organisms. Instead, the telomeres associate with one another, but not with their homologous partners, and this telomere pairing sometimes brings pairs of the telocentric centromeres close together.

**Table 1:** Number of centromere and telomere associated foci in spermatocytes.

		Wild-type		SYCP3 Knockout
No. of nuclei	N	No. CREST foci	No. RAP1 foci	No. CREST foci
Spermatogonia B	100	28.5±1.9	39.2±1.8	32.8±2.0^a^
Pre-leptotene	100	27.1±1.8	39.1±1.6	31.5±1.9^a^
Leptotene	220	36.1±2.6	66.4±1.7	37.0±1.1
Early zygotene	190	36.2±2.1	65.9±1.6*	37.7±1.8^a^
Late zygotene	190	35.1±0.9		38.1±0.9^a^
Pachytene	200	21.0±0.6	38.0±1.5	n.a.
Mid-diplotene	220	22.9±0.7	n.d.	n.a.
Late-diplotene	220	22.7±0.9	n.d.	n.a.
Diakinesis	50	39.5±0.8	n.d.	n.a.

Values for CREST (centromeres) and RAP1 (telomeres) foci are expressed as a mean and corresponding standard deviation.

a Significantly different from wild-type (p≤ 0.05, t test).

*: Mean obtained with values from early and late zygotene.

n.a., not applicable; n.d., not determined.

### Requirement of SYCP1 and SYCP3 in early non-homologous centromere associations

In budding yeast the coupling of non-homologous centromere pairs in very early meiosis (leptotene) requires Zip1 [17], the budding yeast homolog of mouse SYCP1, and does not require other known components of the synaptonemal complex [18]. We asked whether this would be the case in mouse spermatocytes. We performed co-localization experiments to test whether the synaptonemal components SYCP1 or SYCP3 associate with centromere regions in early prophase when telomere associations are occurring (using CREST and SYCP1 or SYCP3 immunostaining). In leptotene we detected no SYCP1 staining, and from early to late zygotene the few traces of foci of SYCP1 mostly did not co-localize with centromeres (1–11.9%, Figure S3). However, SYCP3 is present at early leptotene and, unexpectedly, 97.8% of CREST foci co-localize with SYCP3 signals at this stage (Figure S3). These results indicate another contrast to the situation in yeast; while yeast centromere coupling requires Zip1 but not axial element components, the mouse centromeres at a similar stage of meiosis exhibit co-localization with an axial element component (SYCP3) but not the Zip1 homolog SYCP1.

The period of apparent telomere associations (B type spermatogonia and pre-leptotene, Figure 1) precedes the time at which we first detect clear association of SYCP3 with centromeres (Figure S3). Nonetheless we tested whether SYCP3 might play any role in early centromere association by scoring the numbers of CREST foci in *Sycp3* knockout mice. We observed a significant increase in the number of CREST foci in SYCP3 knockout spermatocytes with respect to wild-type at the stages of B type spermatogonia, pre-leptotene, and zygotene (Figure 1A and Table 1). Whereas the data may suggest that SYCP3 promotes early associations of telomeres (and associated centromeres) the lack of detectable SYCP3 foci in B type spermatogonia and pre-leptotene (result not shown) limits the conclusions.

While we contemplate the possibility of some level of centromere pairing in very early meiosis, the data above can be explained by a period of telomere pairing in early spermatogenesis that brings some centromeres together simply because centromeres are adjacent to telomeres. Furthermore, associations of pericentric heterochromatin clusters that have been observed in spermatogonia could play a role in these associations [19].

In early meiotic prophase we observed an average of 36.1±2.6 CREST foci per leptotene cell (n = 220) and 36.2±2.1 (n = 190) and 35.1±0.9 (n = 190) CREST foci per cell in early and late zygotene respectively. We also observed an average of 66.4±1.7 RAP1 foci per leptotene spermatocyte and 65.9±1.6 RAP1 foci per early/late zygotene spermatocyte. These results are consistent with little or no centromere pairing and a loss of the telomere associations seen in pre-leptotene cells. However, centromeres did appear to cluster primarily on one side of the nucleus as previously described for a bouquet formation [20] (Figure S1, leptotene and zygotene). In pachytene, consistent with completion of homologous chromosome synapsis, we observed an average of 21±0.6 CREST foci per nucleus and 38±1.5 RAP1 foci per nucleus (n = 200) (Figure 1A and 1B and Table 1).

### Persistent pairing of centromeres in late prophase I

We made a remarkable observation when we assayed chromosomes in preserved diplotene cells. The prevailing model for meiotic chromosome behavior is that as cells exit pachytene (towards diplotene) the homologous chromosomes de-synapse and remain joined only at the sites of recombination (chiasmata) which are usually located along their arms. If this model were correct, then we would expect a significant increase in the number of CREST signals as cells move into diplotene. However, in whole cell preparations we observed an average of 22.9±0.7 CREST foci per spermatocyte at mid diplotene (n = 220) and 22.7±0.9 CREST foci per spermatocyte at late diplotene (n = 220) (Figure 1A and Table 1). This important finding indicates that, while the chromosome arms of homologous partners dissociate, their centromeric regions remain paired, just as has been recently reported for yeast [6], [9] and Drosophila females [10].

Given that mouse chromosomes are acrocentric and each centromere is located adjacent to a (short arm) telomere, the question arises as to whether it is an association between telomeres, not centromeres, that is responsible for the reduced number of CREST signals observed in late stages of prophase I. To address this we examined the configuration of individual chromosome pairs in chromosome spread preparations. These preparations are more disruptive than the whole cell preparations used above but offer high resolution views of chromosome configurations (Table 2). Chromosome spreads were stained with SYCP3 to reveal the chromosome axes. In late prophase, nuclear spreads revealed that bivalents frequently exhibited one paired end and one splayed end with internal connections representing chiasma (Figure 1C). For these chromosome figures, tethered by only a central chiasma (for chromosomes experiencing only one exchange event the chiasma is typically located in the central region of the chromosome [21]) we used CREST antibodies to determine whether it was the end with the centromeres that was paired. We observed a strong preferential pairing of the centromeric chromosomal end over the distal telomere (Figure 1C). This supports a model in which the centromeric regions of chromosomes remain paired after chromosome arms dissociate, consistent with Brinkley's first descriptions of mouse centromere behaviors [22]. Intriguingly, we observed that a fraction of homologous chromosomes in the stage of late diplotene show no apparent chiasma (potentially non-exchange) and are only tethered by paired centromeres (Figure 1D, tailed arrows and Figure S4). The frequency of apparent non-exchange chromosomes (4.6%, n = 619) is similar to previous reports (2–4%) [21], [23], [24]. The configuration of the apparent non-exchange chromosomes suggests they have no chiasma, though chromosomes with a single chiasma in the pericentric chromatin would have a similar appearance. We think it is unlikely that many of these chromosomes have pericentric chiasma as genetic exchange is rare in this region [21] and the distribution of MLH1 foci (which mark sites of crossing overs) on our chromosomes indicates that pericentric recombination was rare (Figure S4B). Our observation that centromeres remain paired between apparent non-exchange partners suggests a possible role of centromere pairing in tethering non-exchange chromosomes, which, as described in simple model organisms, may improve their proper segregation.

**Table 2:** Co-localization of synaptonemal complex components and centromeres in diplotene.

	Co-localizing protein	No. of nuclei scored	No. CREST foci	No. co-localizing CREST foci (%)
**Squash**	SYCP1	430	22.8±0.8*	16.2±2.8 (71.1)
**Spread**	SYCP1	124	24.1±2.3	15.5±2.8 (64.3)
	SYCE1	96	24.1±2.3	15.1±2.5 (62.7)
	TEX12	110	24.1±2.3	14.3±1.8 (59.3)

Values corresponding to both surface chromosomes spread and squashed spermatocytes are expressed as the mean and corresponding standard deviation.

*: Mean obtained with values from mid and late diplotene.

In summary, our data suggest that centromere pairing in late prophase I is a conserved component of the meiotic process. The fact that in unicellular model organisms this pairing of centromere regions promotes bi-orientation on the meiotic spindle [5]–[9], suggests that mammals might also use this mechanism to achieve high fidelity meiotic segregation.

### Synapsis is not initiated at centromeres

**Figure 2:**
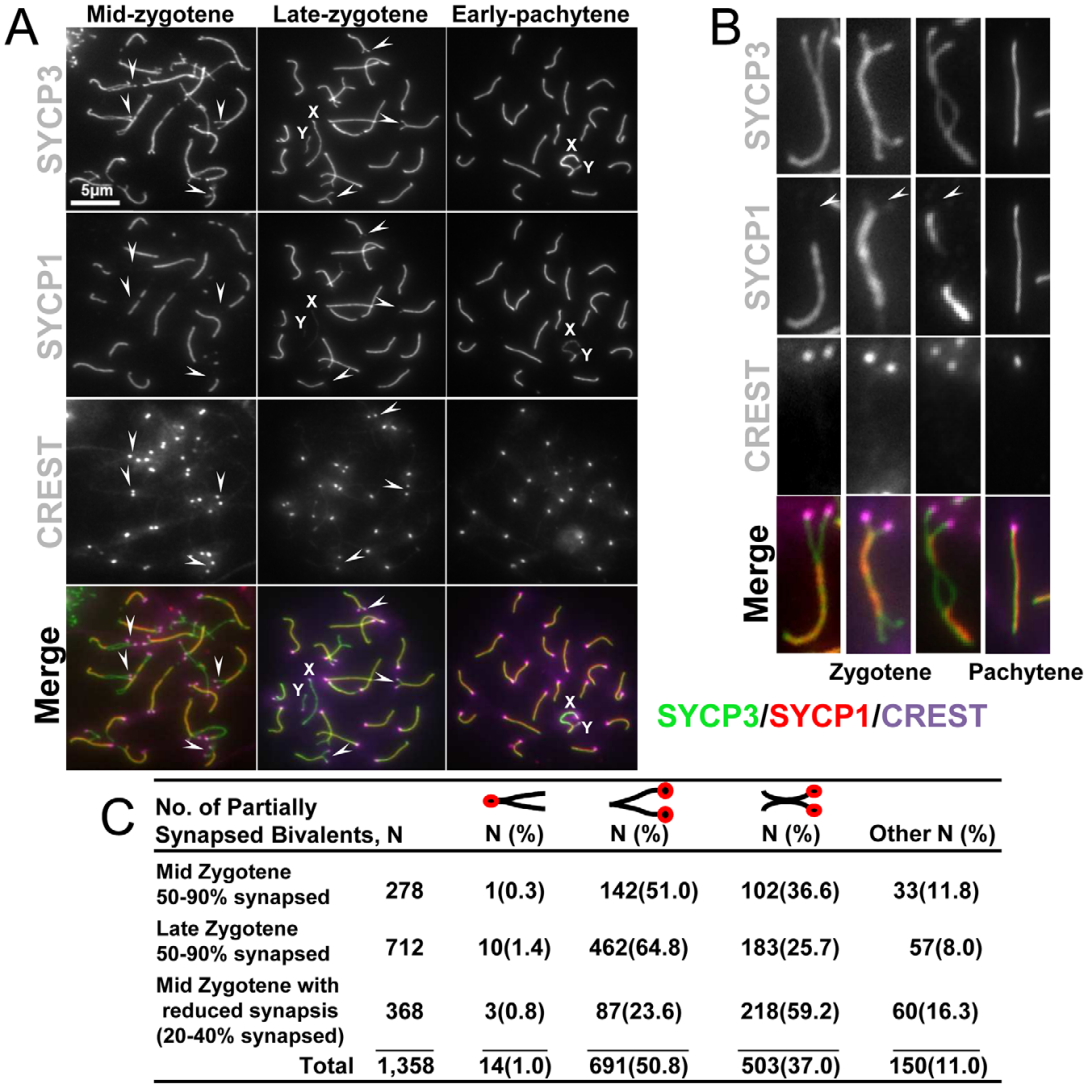
Centromeric regions are the last to associate during homologous chromosome synapsis. (A) Representative surface spread spermatocytes at zygotene and pachytene stages were stained for components of the axial/lateral element (SYCP3), and the transverse filament (SYCP1). CREST staining was used as a marker for centromeres. Arrows indicate unpaired centromeres. X and Y indicate the sex chromosomes. Arrows indicate sites of unpaired centromeres. The scale bar represents 5 µm and corresponds to all images but is only shown in one panel. (B) Magnified zygotene and pachytene chromosomes. Arrows indicate unpaired centromeres (C). Spermatocytes were randomly picked and scored according to extent of pairing and whether regions at the centromere, distal telomere or central chromosome arms were paired. The classification of mid zygotene and late zygotene indicate the average synapsis of all chromosomes in a particular nucleus given the asynchrony of synaptonemal complex assembly. Percentage of synapsis is the total extent of synapsis observed in one individual chromosome. Values are expressed as the total number of chromosomes analyzed at the indicated stage of meiosis. The indicated percentage (%) is the number of chromosomes of an indicated chromosome pairing conformation divided by the total number of chromosomes at an indicated meiotic stage, multiplied by one hundred.

The evaluation of centromere pairing throughout meiosis I, described above, allowed us to address the possibility that centromeres are involved in the initiation of synapsis, as has been suggested from studies in budding yeast [25]. In budding yeast, the centromeres, which are rich in synaptonemal complex components, act as primary initiation sites for chromosome synapsis [25]. We analyzed the partially synapsed chromosomes from spermatocytes in the synapsis process to determine whether centromeres are the first sites of synapsis (Figure 2). Chromosome spreads were stained with antibodies against SYCP3 to visualize the synapsing chromosome axes, SYCP1 to evaluate synaptonemal complex formation and CREST antibodies to reveal the disposition of the centromeres (Figure 2A). The analysis of over one thousand individual partially synapsed chromosomes (Figure 2B) revealed that they fell into several categories. Most common were chromosomes with a single stretch of synaptonemal complex at the non-centromeric end (Figure 2C, 50.8%), and chromosomes with an internal stretch of synaptonemal complex with both ends not yet synapsed (Figure 2C; 37%). In contrast to the situation in budding yeast, chromosomes consistent with initiation of synaptonemal complex at the centromeres (Figure 2C; 1%) were rare. These findings are consistent with observations in many other organisms where synaptonemal complex appears to initiate at internal sites rather than at centromeres. In many instances the internal sites of synapsis initiation appear to be associated with the sites at which recombination initiates (reviewed in [26]).

### Synaptonemal complex is maintained at paired centromeres

Previous studies in yeast and Drosophila have shown that the homologs of mouse SYCP1 are required for late centromere pairing [6], [9], [10]. We therefore tested whether SYCP1 is present at the paired centromeres of mouse spermatocytes at later stages of prophase I using both chromosome spreads and intact cells (squashes of seminiferous tubules). In squashes we observed that approximately 71% of the CREST foci in mid/late diplotene stage spermatocytes co-localized with SYCP1 signals (16.2±2.8 out of 22.8±0.8 CREST foci, n = 430) and observed similar values in chromosome spreads (64.3%, n = 124) (Table 2).

**Figure 3:**
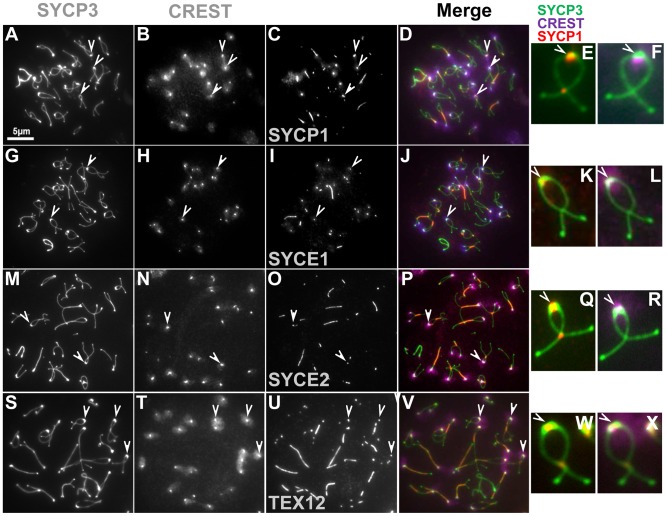
Synaptonemal complex is retained at paired centromeres. (A–F) Representative surface spread spermatocytes at diplotene were immunostained with components of the lateral element (SYCP3) and transverse filaments (SYCP1) of the synaptonemal complex. Spermatocytes were also stained with the components of the central element of the synaptonemal complex SYCE1 (G–L), SYCE2 (M–R) and TEX12 (S–X). Arrowheads indicate paired centromeric regions showing persistent staining for components of the synaptonemal complex. CREST staining (purple) was used as a marker for centromeres. Given the asynchrony of synaptonemal complex transverse filament dissolution in spermatocytes at mid-diplotene some chromosomes may retain threads of SYCP1 in the arms. However, for chromosomes at an advanced stage, SYCP1 is preferentially maintained at paired centromeres. The scale bar represents 5 µm and applies to all images except for magnified images of individual chromosomes.

The co-localization of SYCP1 with centromeres in diplotene, when the synaptonemal complex has disassembled is reminiscent of the situation in yeast where Zip1 localization promotes centromere pairing in late prophase. To test whether there is a correlation between SYCP1 centromeric localization and persistent centromere pairing in late prophase we also analyzed CREST and SYCP1 co-localization on individual chromosome pairs that did or did not exhibit centromere pairing. We observed that while 99.1% chromosomes with paired centromeres exhibited co-localization of SYCP1 with the centromeres, only 8.5% of chromosomes with distal telomere pairing (but not centromere pairing) and only 20.2% of chromosomes held by chromosome arms but not by either centromeres or distal telomeres gave detectable SYCP1 at the unpaired centromeres (Table 3). In sum, the results indicate that as spermatocytes transit through diplotene and metaphase I, SYCP1 is removed from the arms of the chromosomes but remains between paired homologous centromeres (Figure 3A–3F, Table 2 and Table 3). We also analyzed the distribution of components of the central element (SYCE1, SYCE2 and TEX12). As observed for SYCP1, at later stages of prophase I these central element components are removed from chromosome arms but remain at paired homologous centromeres (Figure 3 and Table 2). The analysis of synaptonemal complex proteins distribution on individual chromosome pairs in spread preparations reveal that, as observed for paired centromeres, SYCP1 and components of the central element remain at chiasmata sites (Figure 3). This finding may reveal the existence of a specialized mechanism common for maintenance of synaptonemal complex components at chiasma and paired centromeric areas (see Discussion). Similar observation are made by Qiao et al. in the accompanying study [15].

**Table 3:** SYCP1 association with paired and unpaired centromeres in diplotene.

No. co-localizing CREST-SYCP1				
SC element	Total nuclei, N	N (%) paired centromere	N (%) paired telomere	N (%) interstitial pairing
SYCP1	531	360/363 (99.1)	5/59 (8.5)	22/109 (20.2)

Values are expressed as number of chromosomes showing CREST and SYCP1 co-localization over the total number of chromosomes of each conformation.

Percentages (%) are the number of chromosomes at an indicated chromosome conformation divided by the total number of chromosomes at an indicated meiotic stage, multiplied by one hundred.

**Figure 4:**
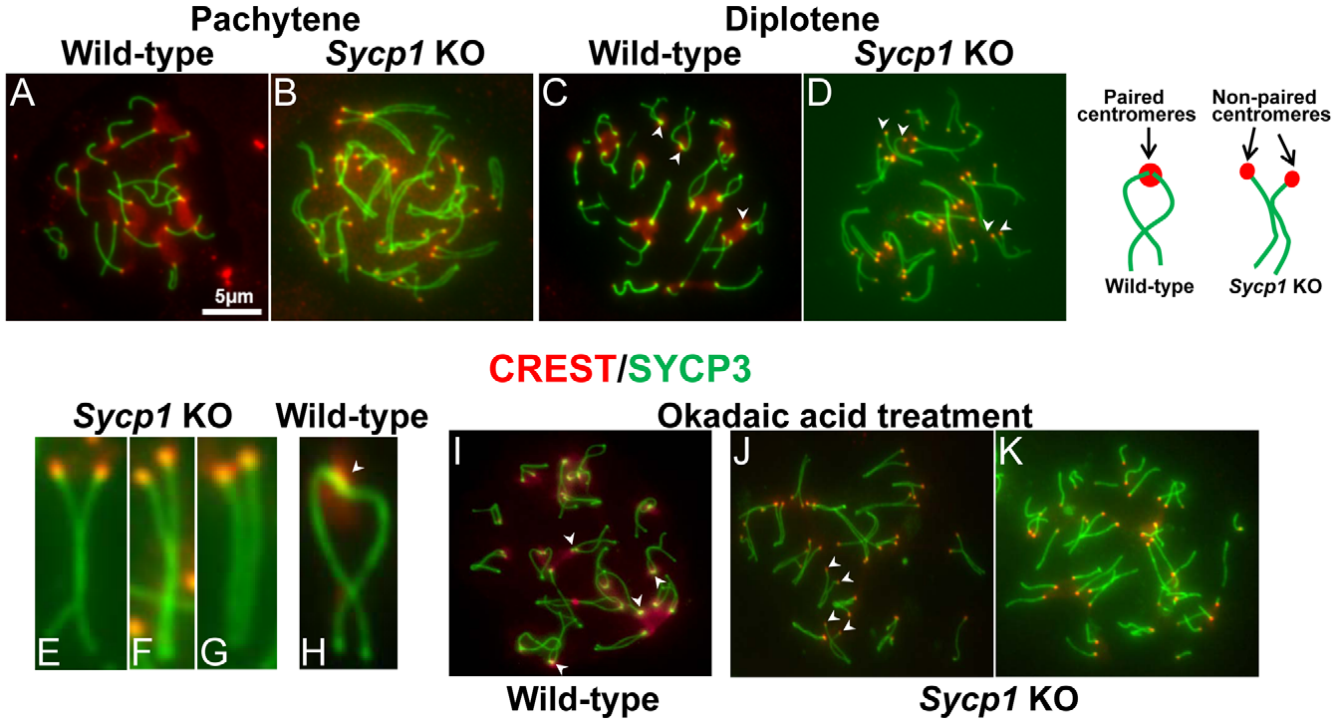
Centromere pairing is absent in *Sycp1* knockout spermatocytes. Surface chromosome spreads of pachytene (A, B) and diplotene (C–H) wild-type and *Sycp1* knockout spermatocytes. Note that centromeres (red) in *Sycp1* knockout spermatocytes are in close proximity but are not paired. In wild-type spreads most centromeric regions remain paired (arrows, C and H), *Sycp1* knockout spermatocytes fail to exhibit paired centromeres (arrows in D). (I–K) Wild-type and *Sycp1* knockout spermatocytes were treated with okadaic acid to induce progression to later stages of prophase I. Note that whereas in wild-type spermatocytes (I) centromeric regions remained paired (arrows), in *Sycp1* knockout spermatocytes (J, K), centromeres are unpaired (J, arrows). The most advanced spermatocytes show an absence of chromosome association (K). The cartoon represents typical homologous chromosomes at mid diplotene exhibiting paired centromeres in wild-type spermatocytes and non-paired centromeres in *Sycp1* knockout mice. Scale bar represents 5 µm and applies to all images except E–H.

To determine whether SYCP1 is necessary for centromere pairing in late prophase we tested whether the absence of SYCP1 leads to loss of centromere pairing in spread chromosomes. In spermatocytes harvested from *Sycp1* knockout mice the homologous chromosomes were aligned in prophase as described previously [12]. However, in contrast to wild-type spermatocytes (CREST foci 21±0.6 (n = 200), homologous centromeres were not paired (*Sycp1* 38.1±1.8 (n = 100). Instead they were located side-by-side but not tightly associated (Figure 4A–4H). Because *Sycp1* knockout results in meiotic prophase arrest, we collected spermatocytes from these mice and drove them *in vitro* through the end of prophase I by treating with okadaic acid [27]–[29]. Here too, without SYCP1, spermatocytes show no association between the homologous centromeres (Figure 4I–4K).

### SYCP1 and SYCP3 are differentially retained at paired centromeres

**Figure 5:**
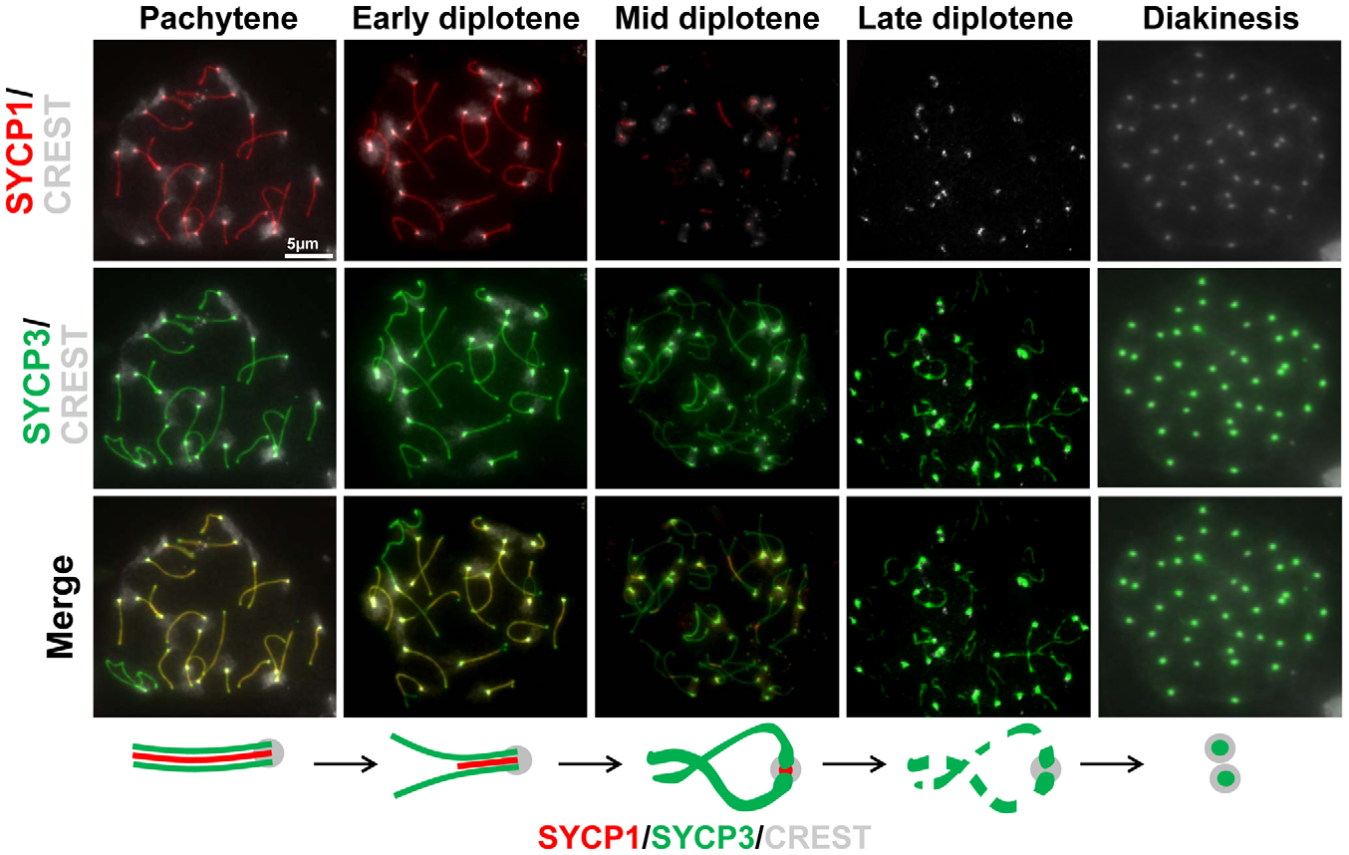
Dynamics of SYCP1 and SYCP3 localization on chromosome arms and centromeres during late prophase I. Surface chromosome spreads were simultaneously stained for SYCP1 and SYCP3 in order to determine their relative timing of disassembly/dissociation from the synaptonemal complex at later stages of prophase I. The cartoon summarizes the observations. The scale bar represents 5 µm and applies to all panels.

**Figure 6:**
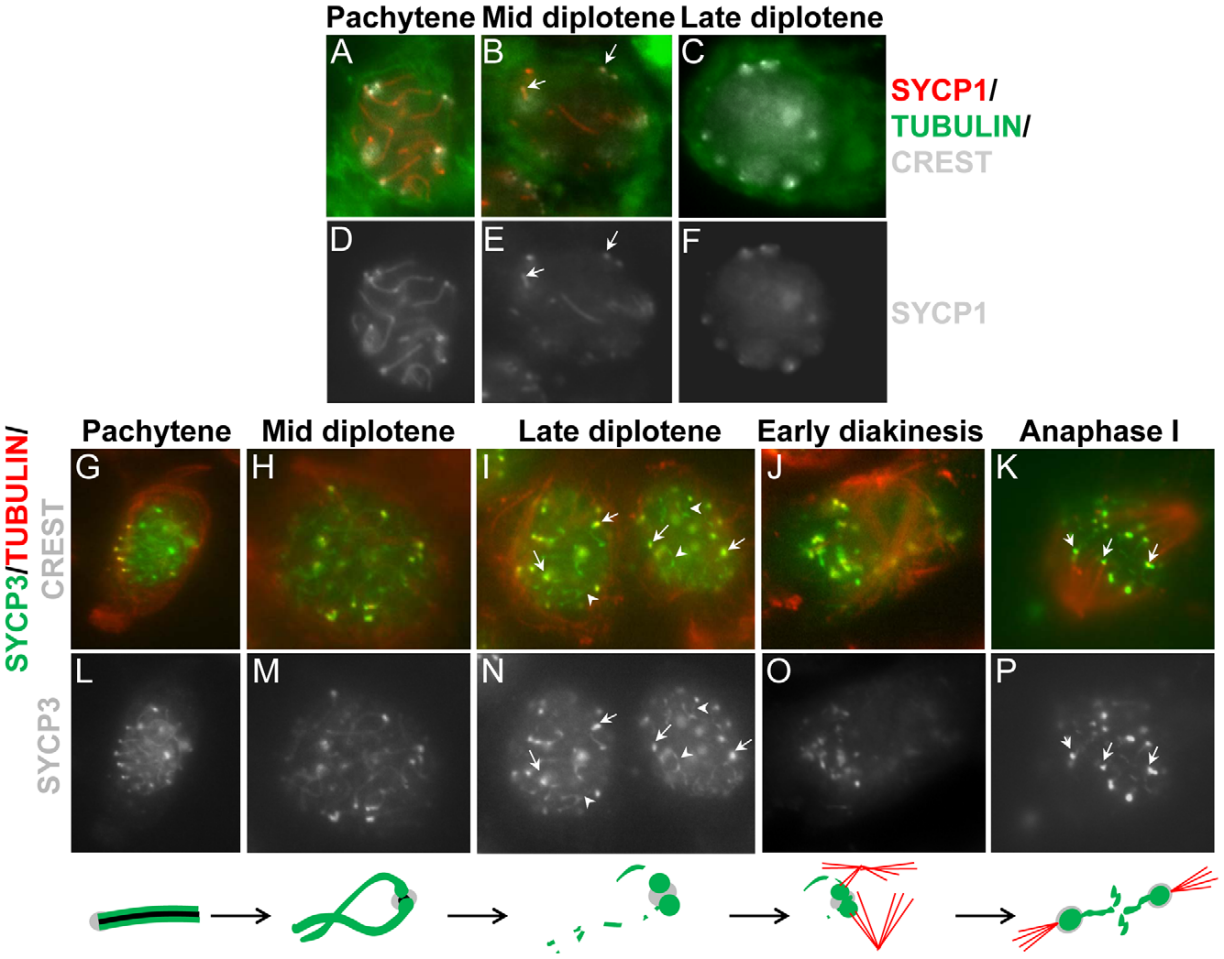
SYCP1 and SYCP3 are differentially retained on chromosomes as cells progress through meiosis I. Imaging of whole cells was used to evaluate the distribution of SYCP1, SYCP3 and tubulin. (A–F) After removal from chromosome arms SYCP1 is preferentially retained at paired centromeres in spermatocytes until mid diplotene (panel B, arrows). In a subsequent step (panel c, late diplotene) SYCP1 is removed from paired centromeres before or at nuclear envelope break down. (G–P) SYCP3 is removed from chromosome arms in diplotene but is maintained at paired centromeres until anaphase I. Note the reduction of SYCP3 at chromosome arms (panel i, arrowheads) and accumulation at centromeres after late diplotene (panel i, tailed arrows). After nuclear envelope break down and assembly of the spindle a fraction of SYCP3 bridges the centromeres at anaphase I (panels K, white arrows). Single fluorescent channel images for SYCP1 (panels D–F) and SYCP3 (panels L–P) is also shown. The cartoon summarizes the observations. SYCP1 is shown in black. The scale bar represents 5 µm and applies to all panels.

Our results showing the retention of synaptonemal complex components at the centromeres in late prophase (Figure 3 and Table 2), together with the previous demonstration that the lateral element protein SYCP3 remains at centromeres after lateral element disassembly [29]–[32] suggests that these proteins may play important roles at meiotic centromeres. To evaluate the relative behaviors of SYCP3 and synaptonemal complex central region components we first monitored the kinetics of SYCP1 and SYCP3 sub-chromosomal distribution relative to one another in late prophase I on spread chromosomes (Figure 5). SYCP3 and SYCP1 are both retained at centromeres in diplotene following synaptonemal complex disassembly but SYCP1 was not detectable by late diplotene and diakinesis. In contrast, as described previously, SYCP3 persists until metaphase I (Figure 5) [29]–[31]. If centromere pairing, mediated by either of these proteins promotes bi-orientation on the emerging spindle, SYCP1 and SYCP3 might persist at the centromeres as microtubule attachments begin to be established. To examine this possibility, indirect immunofluorescence was used to monitor the distribution of SYCP1 and SYCP3, in cells from meiotic squashes, relative to the emergence of the spindle that is formed as cells progress from late prophase to metaphase. Cell squash preparations were used as these, in contrast to surface chromosome spreads, allow staining of tubulin, which can be used as a marker for nuclear envelope dissolution and meiotic spindle assembly. After removal from chromosome arms SYCP1 is preferentially retained, in spermatocytes at mid diplotene, at punctate foci that correspond to centromeres (as was shown in the chromosome spreads in Figure 5). These foci are indicated by white arrows in the single plane images of the stained cells (shown in Figure 6b). In a subsequent stage (late diplotene) SYCP1 becomes undetectable before nuclear envelope break down and before any clear assembly of microtubules around the chromosomes occurs (Figure 6C). In contrast, SYCP3 is removed primarily from the arms of chromosomes by diplotene but is maintained at paired centromeres as microtubules begin to assemble around the chromosomes. SYCP3 remains associated with the sister centromeres until anaphase I (Figure 6G–6K). Whereas persistence of SYCP3 has been interpreted as evidence for a role of SYCP3 in the diplotene-metaphase I transition [29]–[31], some observations argue against this hypothesis [33]. In summary, we observed striking differences in the timing of disappearance/relocation of the SYCP1 and SYCP3 synaptonemal complex proteins from paired homologous centromeres. Both proteins persist at or between centromeres following synaptonemal complex disassembly. However, most SYCP1 leaves the centromeres in late diplotene while SYCP3 persists through the stages of microtubule attachment to kinetochores and meiosis I separation of the homologous partners.

## Discussion

### Homologous centromere pairing in mouse meiosis

Active pairing of meiotic centromeres has been observed in a variety of organisms and occurs at two different stages of meiotic prophase I. Pairing of centromeres by apparently homology-independent processes in early meiosis has been seen in a variety of organisms, and in yeast has been termed centromere coupling [17]. At present, the function of centromere coupling is not understood. However, in budding yeast, it is mediated by the synaptonemal complex central element component Zip1 and does not require other proteins that normally participate in synaptonemal complex assembly [18]. In mouse, we observed that spermatocytes do not exhibit prolonged period of nearly complete centromere coupling (Figure 1A and Table 1). Surprisingly, we found evidence for a period in which telomeres appear to be arranged in pairs or small groups independent of homology. Our data can be explained by a period of telomere pairing that brings some centromeres together simply because centromeres are adjacent to telomeres [19]. If there is a period of nearly complete centromere pairing in early prophase mouse spermatocytes, as is seen in some other organisms [2], [17], [34]–[39], such pairing must be transient.

**Figure 7:**
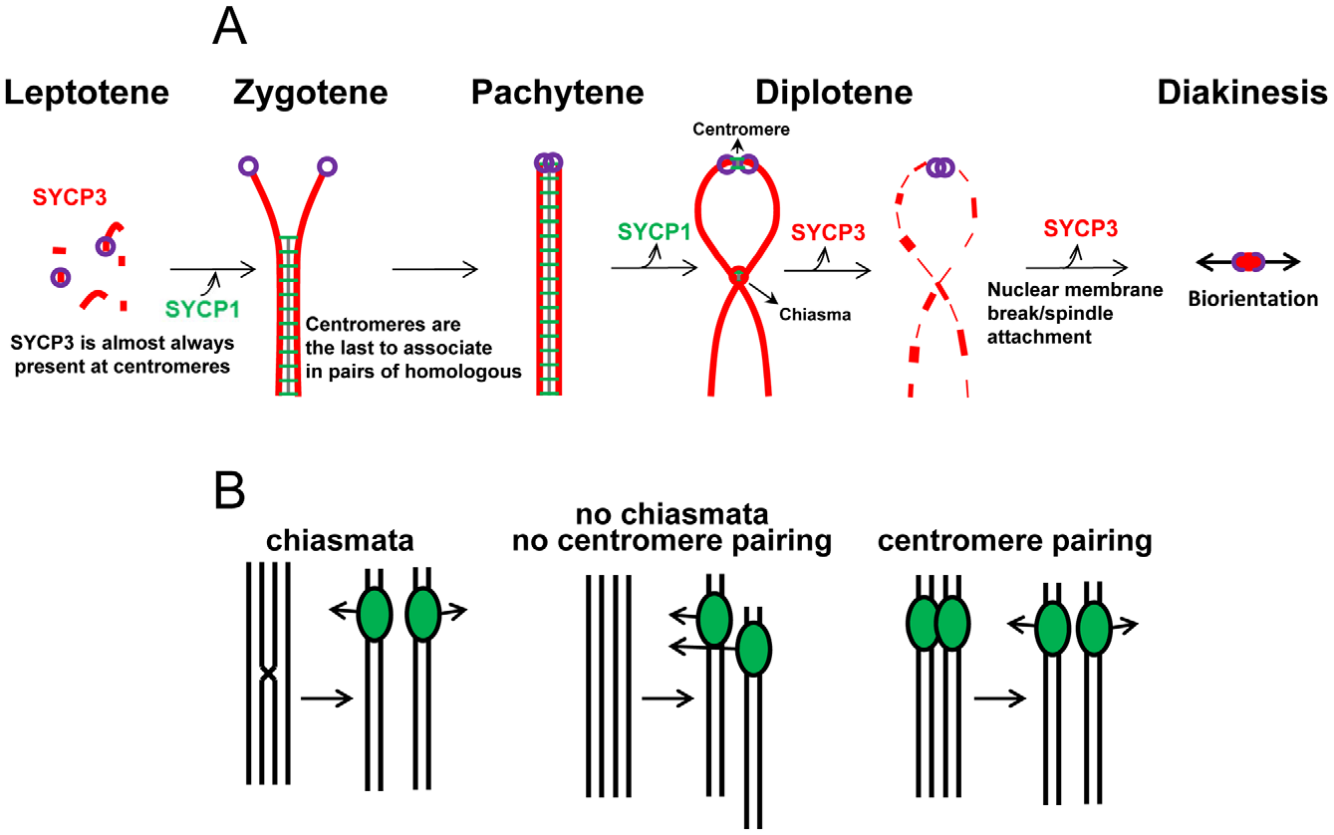
The coordination of centromere pairing and chromosome segregation at meiosis I. (A) Model for the role of SYCP1 and SYCP3 in centromere pairing. SYCP1 and SYCP3 play different roles in meiotic chromosome segregation while cohering homologous centromeres. (B) Based on the similarities between mice and other model organism such budding yeast and Drosophila, in which a correlation between late centromere pairing and improved segregation of homologous chromosomes has been demonstrated, we propose that in the absence of genetic exchange, centromere pairing holds homologous centromeres together and facilitates orientation of the homologous chromosomes for movement to the opposite poles during meiosis I division.

In a variety of organisms, pairing between homologous centromeres in late prophase has been implicated in promoting proper meiosis I segregation. Studies focused on the meiotic behavior of non-exchange homologous chromosomes in Drosophila females strongly suggests that pairing between regions of pericentric heterochromatin allow the chromosome pairs to attach to the spindle in a bipolar fashion that mediates disjunction at meiosis I [7], [8]. Similarly, in yeast, pairing between the homologous centromeres promotes bi-orientation of both non-exchange partners and homologous partners that have experienced crossovers [2], [5], [9]. In both budding yeast and Drosophila oocytes, centromeric associations in late meiotic prophase require components of the synaptonemal complex, which persist at the centromeres when the rest of the synaptonemal complex is disassembled [2], [10]. Our results demonstrating centromere pairing in later stages of prophase, and the participation of SYCP1 and SYCP3 in the process, parallel those reported in budding yeast and Drosophila. In those organisms centromere pairing significantly improves segregation of non-exchange chromosomes and may well improve the segregation fidelity of exchange chromosomes as well. Based on these similarities, we propose that also in mice centromere pairing may promote proper segregation of homologous chromosomes. Indeed, we have observed that SYCP1 and SYCP3 may play separate roles in keeping homologous centromeres together at different stages of prophase I. The stage at which SYCP1 is lost from the arms and maintained at the homologous centromeres is co-incident with the time that certain kinetochore-specific proteins re-assemble onto the chromosomes and load at centromeres [6], [10], [14] (Figure S5). We propose that SYCP1 may act at the G2/M transition by contributing to the assembly of kinetochores that will promote meiosis I specific patterns of segregation (Figure 7). Although SYCP1 is not detectable between paired homologous centromeres at late diplotene, directly or indirectly it contributes to this pairing, as centromere pairing is not seen in SYCP1 knockout mice. Thus, the presence of SYCP1 at centromeres in earlier stages of meiotic prophase may be a prerequisite for establishing paired structures that persist into diplotene after SYCP1 is normally removed from centromeres, thus SYCP1 may be required more for the establishment than the maintenance of centromere pairing. In late diplotene, after removal of central region proteins between homologous centromeres, SYCP3 remains only at centromeres. We propose that SYCP3 maintains centromere pairing and contributes to bi-orientation of homologous centromeres and to spindle attachment that prepares the homologous chromosomes for the first meiotic division (Figure 7).

### Mechanism of regulation for centromere pairing

As spermatocytes transit from pachytene (complete synapsis) to diplotene, synaptonemal complex proteins are lost from the chromosome arms but persist at the paired centromeres (Figure 3). Phosphorylation of synaptonemal complex components correlates with their removal/dissociation from chromosomes [29], [40], and Polo-like kinase (PLK1) promotes synaptonemal complex disassembly in yeast and mice [29], [41], [42]. Our results suggest that removal of synaptonemal components is mechanistically different at the arms compared to the centromere. This might be explained if centromeres are paired by structures distinct from the synaptonemal complex. However, we saw no evidence of this since all the elements of the synaptonemal complex are present at paired centromeres (Figure 3). In an alternative hypothesis we propose that the synaptonemal complex proteins are in the same structures on arms and centromeres, but are protected from removal at the centromeres during disassembly. The existence of a specialized mechanism for maintenance of synaptonemal complex proteins at centromeric regions has support in previous reports. Here, Shugoshin (SGO2)/PP2A was shown to protect cohesin complexes at centromeres, when arm cohesins are lost during the metaphase I to anaphase I transition, by antagonizing the action of kinases [41], [43]–[45]. Indeed, SYCP1 and SYCP3 have potential PLK1 phosphorylation sites, and PLK1 only loads on meiotic centromeres at later stages of meiosis I coincident with the time when SYCP1 and SYCP3 are retained at centromeres [14] (Figure S5). Based on these results, we hypothesize that a dynamic interplay between PLK1 and SGO2-PP2A controls SYCP1 and SYCP3 phosphorylation, which then regulates retention of these proteins at centromeres with consequent regulation of centromere pairing.

## Materials and Methods

### Mice

Mice used in this study were as follows: Wild-type (C57BL/6), *Sycp3* knockout [46], *Sypc*1 knockout [12]. Experiments conformed to relevant regulatory standards and were approved by the IACUC Institutional Animal Care and Use Committee.

### Spermatocyte squash preparation

Adult male C57BL/6 mice 4–8 weeks old were euthanized by CO_2_ inhalation followed by cervical dislocation. The testes were then removed and detunicated, and seminiferous tubules processed for squashing. For squashing, we followed a technique previously described [14]. Briefly, seminiferous tubules were fixed in freshly prepared 2% formaldehyde in 1× PBS containing 0.1% Triton X-100. After 5 min, several seminiferous tubule fragments were placed on a slide and squashed, and the coverslip removed after freezing in liquid nitrogen. Samples were washed with 1× PBS and stored up to 4 days before use.

### Cytology

We employed established experimental approaches for the visualization of chromosomes in both structurally-preserved nuclei (seminiferous tubules squashes) and surface spreads [47]. Incubations with primary antibodies were carried out for 1 h at room temperature in 1× PBS plus BSA 2%. To detect SYCP1 and SYCP3 we used polyclonal rabbit antibody raised against mouse SYCP1 at 1∶200 dilution (Novus Biologicals) and polyclonal mouse antibody raised against mouse SYCP3 at 1∶300 dilution (ABcam). Centromeres were detected using the human centromere protein antibody (CREST, Antibody Incorporated) at 1∶50 dilution. Other primary antibodies used in this study were as follows: polyclonal mouse antibody raised against human AURORA B at 1∶50 dilution, polyclonal rabbit antibody raised against human PLK 1 at 1∶50 dilution (Upstate), polyclonal mouse antibody raised against human BUB1 at 1∶50 dilution (a gift from S. Taylor [48]), polyclonal mouse antibody raised against human CDC20 at 1∶50 dilution (a gift from J. Weinstein [49]). Following three washes in 1× PBS, slides were incubated for 1 h at room temperature with secondary antibodies. A combination of fluorescein isothiocyanate (FITC)-conjugated goat anti-rabbit IgG (Jackson laboratories) with Rhodamine-conjugated goat anti-mouse IgG and Cy5-conjugated goat anti-human IgG each diluted 1∶250 were used for simultaneous triple immunolabeling. Slides were subsequently counterstained for 3 min with 2 µg/ml DAPI containing Vectashield mounting solution (Vector Laboratories) and sealed with nail varnish. For CREST and RAP1 focus counts, nuclei were staged using the extent of SYCP3 staining and synapsis as markers for meiotic prophase progression. Quantification of co-localizing CREST with SYCP1, SYCE1 and TEX12 were carried out by superimposing images of the corresponding fluorescent channels in a single plane image. When two CREST foci were observed together without any gap in between them, we considered those signals as two distinct foci. We use Axiovision SE 64 (Carl Zeiss, inc.) for imaging acquisition and processing. Statistical tests were as described in the table legend.

## Supporting Information

Figure S1 Identification of sub-stages of meiotic prophase I in squash preparations of mouse seminiferous tubules. Labeled are DNA (DAPI, grey), meiotic chromosome axes (SYCP3, green), the central region of the synaptonemal complex (SYCP1, red) and centromeres (CREST, yellow). EDU staining (green, 3^rd^ row) was used as a marker for DNA synthesis. Single-plane fluorescent images from 4-color stacks are shown at the nuclear equator. Nucleus size and distinctive patterns of heterochromatin shown by DNA fluorescence correlate with the sub-stage of meiotic prophase in wild-type specimens. Sgt B, B type spermatogonia. Pre-Lept. (1), pre-leptotene type 1. Pre-Lept. (2), pre-leptotene type 2. Two types of cells at pre-leptotene stage are defined according to intensity and distribution of EDU signals. Scale bar represents 5 µm and applies to all panels.
(PPTX)

Figure S2 A fraction of meiocyte chromosomes at pre-meiotic S-phase exhibit centromeres and proximal telomeres non-homologously clustered or paired. (A) Fluorescence in situ hybridization with a chromosome VIII region-specific probe showing examples of both paired and non-paired homologous chromosomes in tested nuclei. FISH experiments were performed as describe in Text S1. (B) Quantitation of pairing of the homologous FISH-tagged chromosome VIII region in B type spermatogonia (n = 35) and spermatocytes at pre-leptotene (n = 49), leptotene (n = 31), zygotene (n = 31), pachytene (n = 35), mid-diplotene (n = 50), late-diplotene (n = 30) and diakinesis (n = 25). The values agree with a previous analysis of wild-type spermatocytes [19]. Distances between fluorescent foci in diakinesis were shorter with respect to those detected in pre-leptotene. This may reflect differences in chromosome condensation and nuclear localization. Non-paired is defined as two discreet fluorescent signals separated by a gap.
(PPTX)

Figure S3 SYCP3 but not SYCP1co-localizes with CREST signals in leptotene and zygotene spermatocytes. (A) Spermatocytes were stained for SYCP3 (a component of the lateral element), SYCP1 (transverse filaments) and CREST (a centromere marker). Arrows indicate some sites of CREST and SYCP3 co-localization. Scale bar represents 5 µm and applies to all panels. (B) Spermatocytes at stages from early leptotene to pachytene were randomly picked and scored for the number of CREST foci and co-localization of CREST with SYCP3 and SYCP1 signals. Values are expressed as mean ± standard deviation for each group of chromosomes in the indicated meiotic stage. n.a, not applicable.
(PPTX)

Figure S4 A fraction of chromosomes in the stage of late diplotene are only tethered by paired centromeres. (A) Quantification of chromosomes with no apparent chiasmata and tethered by paired centromeres. Chromosomes experiencing a central chiasmata and paired centromeres are shown for comparison. (B) Distribution of crossing over on autosomes experiencing one MLH1 focus. Spread pachytene nuclei were stained for SYCP3, MLH1 and CREST (to detect pericentromeric chromatin), and the positions of MLH1 foci were measured relative to centromeres and expressed as percentage of the synaptonemal complex length. Note that only two out of 450 total chromosomes scored show a MLH1 focus at the centromeric region.
(PPTX)

Figure S5 SYCP1 and SYCP3 are selectively localized at paired centromeres coincident with the time at which kinetochore-specific proteins begin loading chromosomes. Indirect immunofluorescence was used to monitor the distribution of several kinetochore-specific proteins, SYCP1 (green) and SYCP3 (green in PLK1 and INCENP columns) in surface spread spermatocytes progressing from late prophase to late diplotene. Known kinetochore components AURORA B, BUB1, phosphorylated histone H3, CDC20, PLK1, and INCENP are shown in red. CREST is shown in grey. Scale bar represents 5 µm and applies to all panels.
(PPTX)

Text S1 Protocol for DNA FISH on spermatocyte squash preparations.
(DOCX)
