# Supplementary material for: Synaptonemal Complex Components Persist at Centromeres and Are Required for Homologous Centromere Pairing in Mouse Spermatocytes
Source: PLoS Genet. 2012 Jun 28;8(6):e1002701. doi: 10.1371/journal.pgen.1002701 (PMC3386160; doi:10.1371/journal.pgen.1002701)
Supplement: Figure S1 — Identification of sub-stages of meiotic prophase I in squash preparations of mouse seminiferous tubules. Labeled are DNA (DAPI, grey), meiotic chromosome axes (SYCP3, green), the central region of the synaptonemal complex (SYCP1, red) and centromeres (CREST, yellow). EDU staining (green, 3rd row) was used as a marker for DNA synthesis. Single-plane fluorescent images from 4-color stacks are shown at the nuclear equator. Nucleus size and distinctive patterns of heterochromatin shown by DNA fluorescence correlate with the sub-stage of meiotic prophase in wild-type specimens. Sgt B, B type spermatogonia. Pre-Lept. (1), pre-leptotene type 1. Pre-Lept. (2), pre-leptotene type 2. Two types of cells at pre-leptotene stage are defined according to intensity and distribution of EDU signals. Scale bar represents 5 µm and applies to all panels. (PPTX) [file pgen.1002701.s001.pptx]

## Slide 1
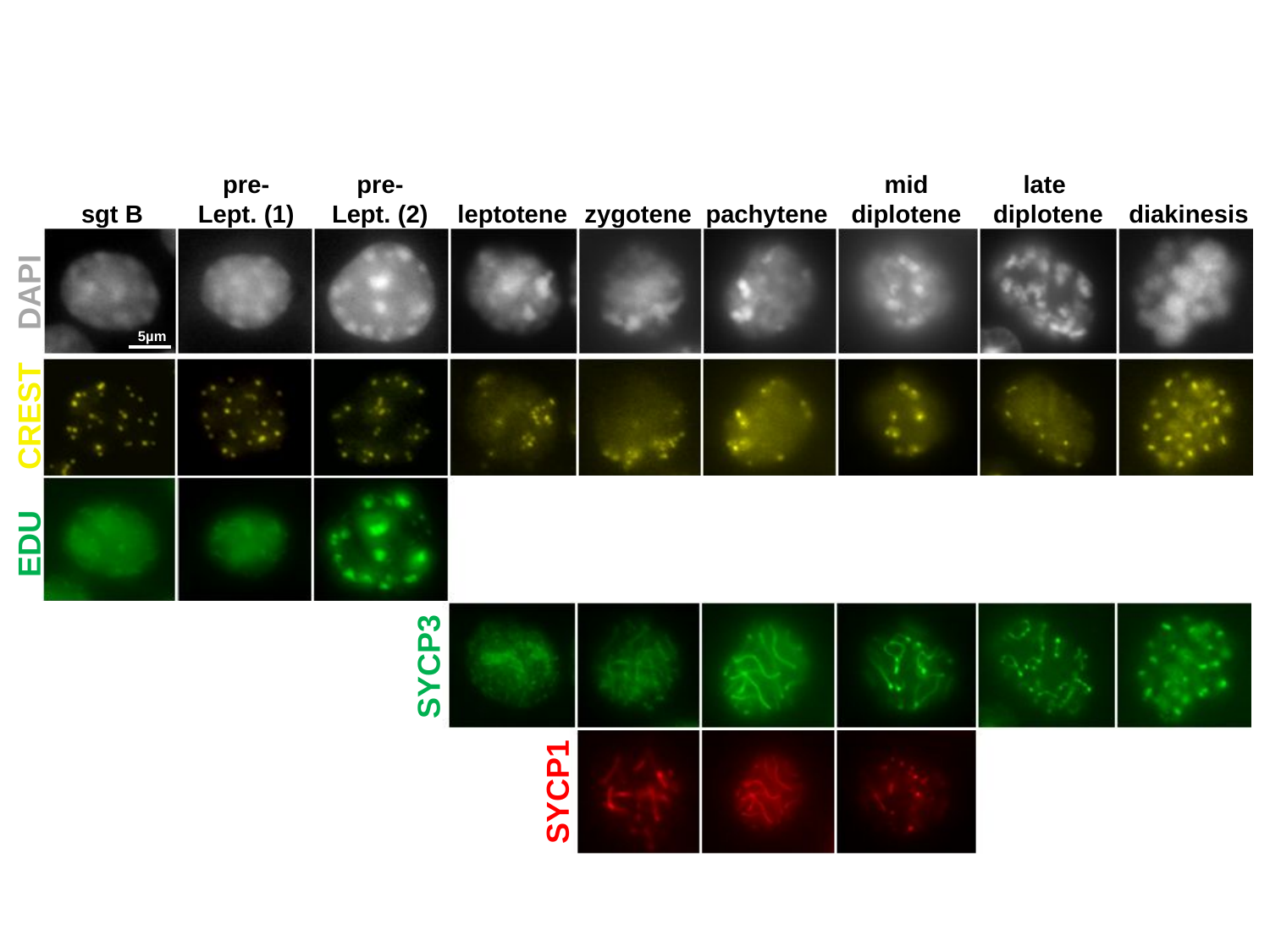

pre-
Lept. (1)
pre-
Lept. (2)
mid
diplotene
late
diplotene
sgt B
leptotene
zygotene
pachytene
diakinesis
DAPI
5µm
5µm
CREST
EDU
SYCP3
SYCP1
