# Supplementary material for: Synaptonemal Complex Components Persist at Centromeres and Are Required for Homologous Centromere Pairing in Mouse Spermatocytes
Source: PLoS Genet. 2012 Jun 28;8(6):e1002701. doi: 10.1371/journal.pgen.1002701 (PMC3386160; doi:10.1371/journal.pgen.1002701)
Supplement: Figure S2 — A fraction of meiocyte chromosomes at pre-meiotic S-phase exhibit centromeres and proximal telomeres non-homologously clustered or paired. (A) Fluorescence in situ hybridization with a chromosome VIII region-specific probe showing examples of both paired and non-paired homologous chromosomes in tested nuclei. FISH experiments were performed as describe in Text S1. (B) Quantitation of pairing of the homologous FISH-tagged chromosome VIII region in B type spermatogonia (n = 35) and spermatocytes at pre-leptotene (n = 49), leptotene (n = 31), zygotene (n = 31), pachytene (n = 35), mid-diplotene (n = 50), late-diplotene (n = 30) and diakinesis (n = 25). The values agree with a previous analysis of wild-type spermatocytes [19]. Distances between fluorescent foci in diakinesis were shorter with respect to those detected in pre-leptotene. This may reflect differences in chromosome condensation and nuclear localization. Non-paired is defined as two discreet fluorescent signals separated by a gap. (PPTX) [file pgen.1002701.s002.pptx]

## Slide 1
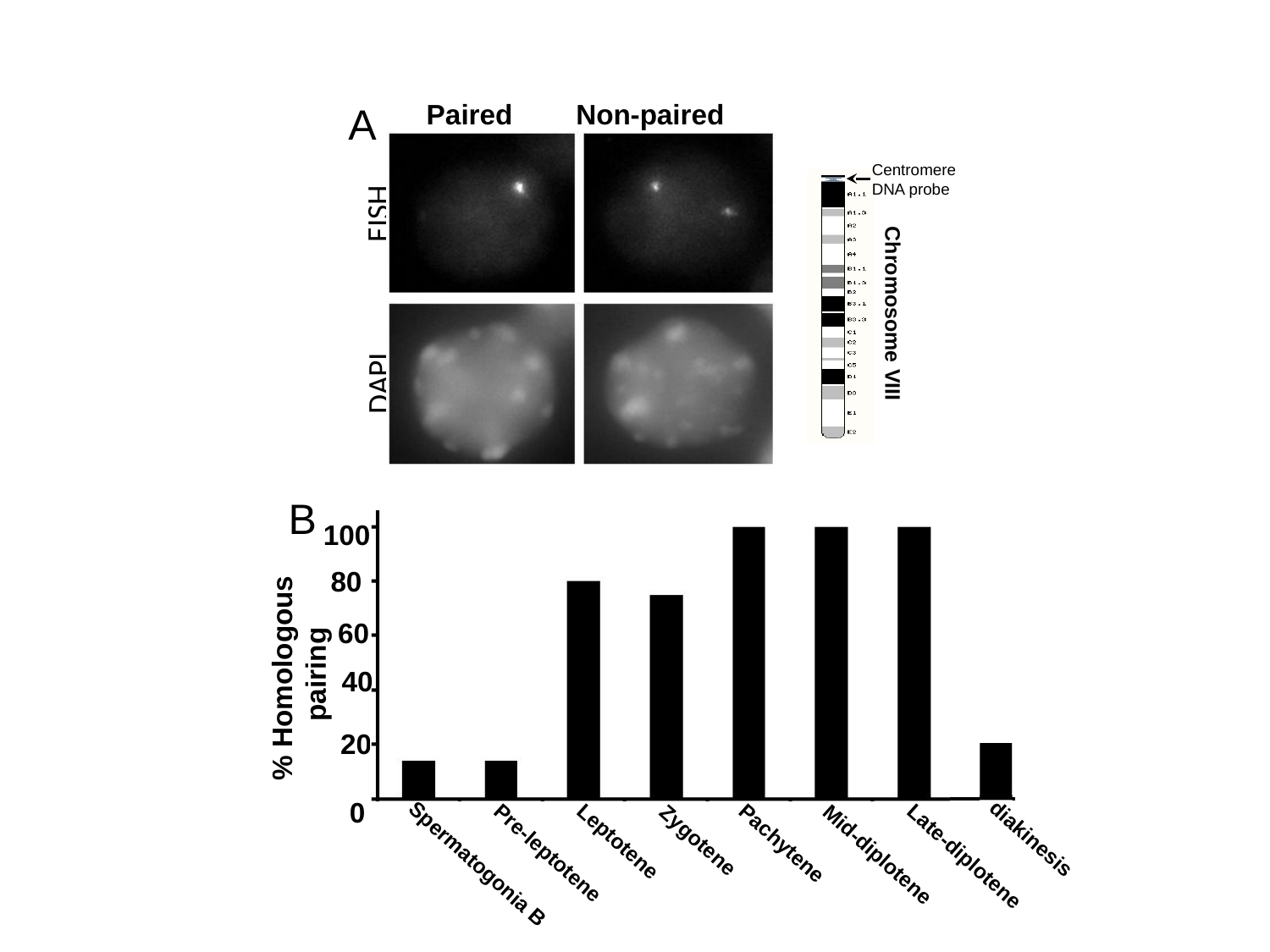

Paired
Non-paired
A
Centromere
DNA probe
Chromosome VIII
FISH
DAPI
B
100
80
60
% Homologous
 pairing
40
20
0
diakinesis
Zygotene
Leptotene
Pachytene
Pre-leptotene
Mid-diplotene
Late-diplotene
Spermatogonia B
