# Supplementary material for: Synaptonemal Complex Components Persist at Centromeres and Are Required for Homologous Centromere Pairing in Mouse Spermatocytes
Source: PLoS Genet. 2012 Jun 28;8(6):e1002701. doi: 10.1371/journal.pgen.1002701 (PMC3386160; doi:10.1371/journal.pgen.1002701)
Supplement: Figure S3 — SYCP3 but not SYCP1co-localizes with CREST signals in leptotene and zygotene spermatocytes. (A) Spermatocytes were stained for SYCP3 (a component of the lateral element), SYCP1 (transverse filaments) and CREST (a centromere marker). Arrows indicate some sites of CREST and SYCP3 co-localization. Scale bar represents 5 µm and applies to all panels. (B) Spermatocytes at stages from early leptotene to pachytene were randomly picked and scored for the number of CREST foci and co-localization of CREST with SYCP3 and SYCP1 signals. Values are expressed as mean ± standard deviation for each group of chromosomes in the indicated meiotic stage. n.a, not applicable. (PPTX) [file pgen.1002701.s003.pptx]

## Slide 1
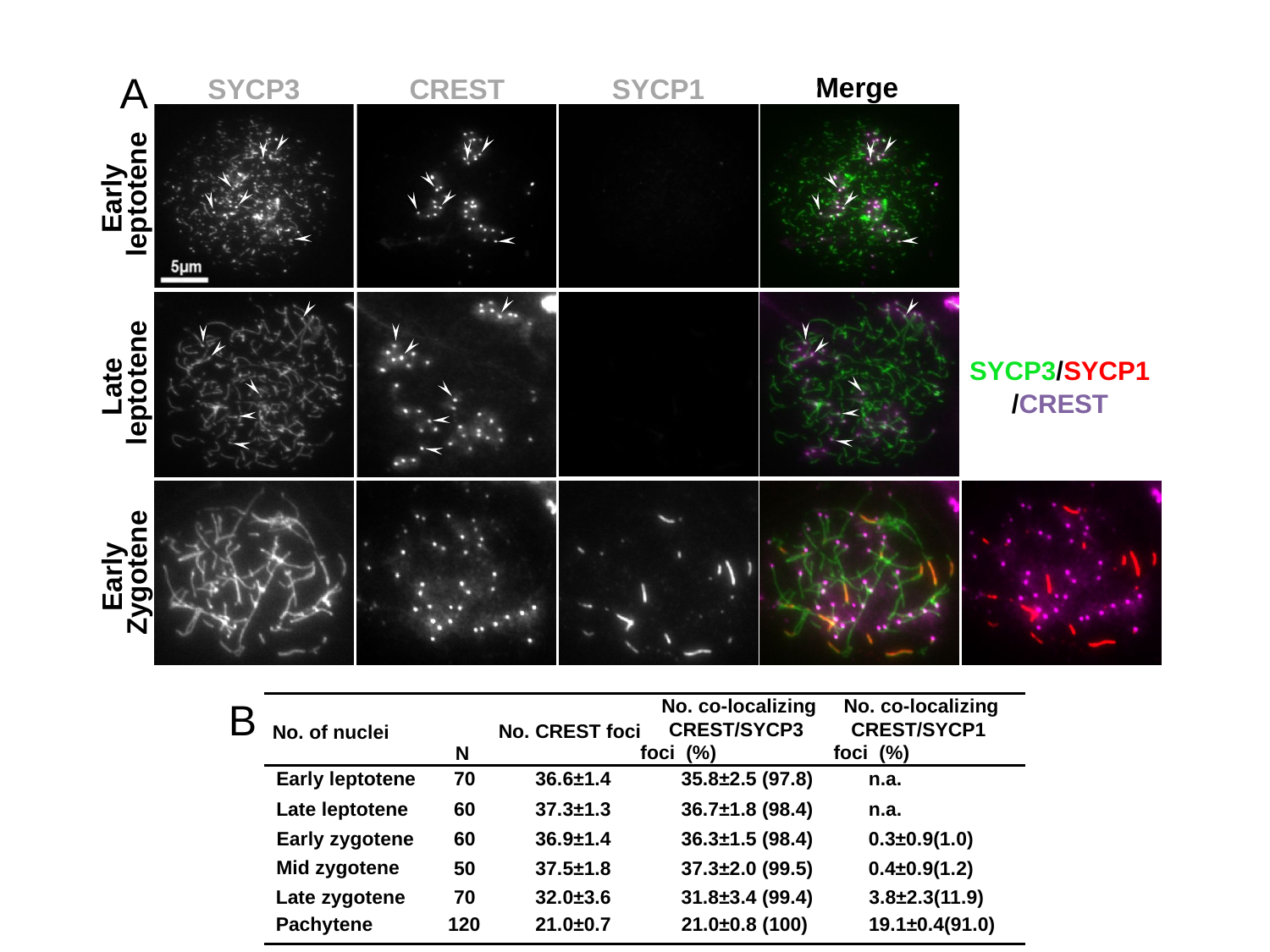

A
Merge
SYCP3
CREST
SYCP1
Early
leptotene
SYCP3/SYCP1
/CREST
Late
leptotene
Early
Zygotene
B
No. co-localizing CREST/SYCP3
foci (%)
No. co-localizing CREST/SYCP1
foci (%)
No. CREST foci
No. of nuclei
N
70
36.6±1.4
35.8±2.5 (97.8)
n.a.
Early leptotene
60
37.3±1.3
36.7±1.8 (98.4)
n.a.
Late leptotene
60
36.9±1.4
36.3±1.5 (98.4)
0.3±0.9(1.0)
Early zygotene
50
37.5±1.8
37.3±2.0 (99.5)
0.4±0.9(1.2)
Mid zygotene
70
32.0±3.6
31.8±3.4 (99.4)
3.8±2.3(11.9)
Late zygotene
120
21.0±0.7
21.0±0.8 (100)
19.1±0.4(91.0)
Pachytene
