# Supplementary material for: Synaptonemal Complex Components Persist at Centromeres and Are Required for Homologous Centromere Pairing in Mouse Spermatocytes
Source: PLoS Genet. 2012 Jun 28;8(6):e1002701. doi: 10.1371/journal.pgen.1002701 (PMC3386160; doi:10.1371/journal.pgen.1002701)
Supplement: Figure S4 — A fraction of chromosomes in the stage of late diplotene are only tethered by paired centromeres. (A) Quantification of chromosomes with no apparent chiasmata and tethered by paired centromeres. Chromosomes experiencing a central chiasmata and paired centromeres are shown for comparison. (B) Distribution of crossing over on autosomes experiencing one MLH1 focus. Spread pachytene nuclei were stained for SYCP3, MLH1 and CREST (to detect pericentromeric chromatin), and the positions of MLH1 foci were measured relative to centromeres and expressed as percentage of the synaptonemal complex length. Note that only two out of 450 total chromosomes scored show a MLH1 focus at the centromeric region. (PPTX) [file pgen.1002701.s004.pptx]

## Slide 1
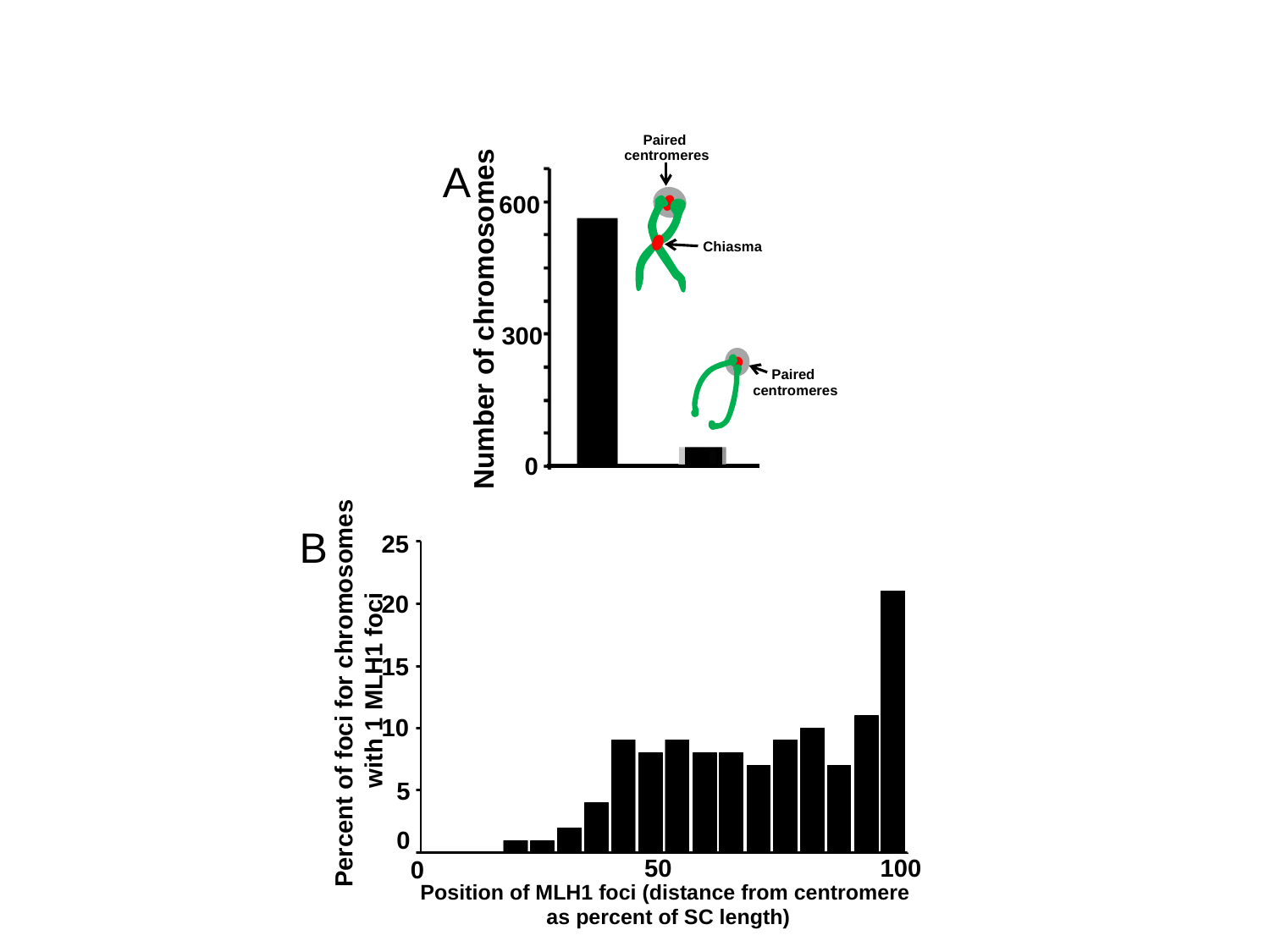

Paired
centromeres
Chiasma
A
600
Number of chromosomes
300
Paired
centromeres
0
B
25
25
20
15
Number of bivalents per cell
10
5
0
Wt
Hfm1
20
15
Percent of foci for chromosomes
with 1 MLH1 foci
10
5
0
50
100
0
Position of MLH1 foci (distance from centromere
as percent of SC length)
