# Supplementary material for: Synaptonemal Complex Components Persist at Centromeres and Are Required for Homologous Centromere Pairing in Mouse Spermatocytes
Source: PLoS Genet. 2012 Jun 28;8(6):e1002701. doi: 10.1371/journal.pgen.1002701 (PMC3386160; doi:10.1371/journal.pgen.1002701)
Supplement: Figure S5 — SYCP1 and SYCP3 are selectively localized at paired centromeres coincident with the time at which kinetochore-specific proteins begin loading chromosomes. Indirect immunofluorescence was used to monitor the distribution of several kinetochore-specific proteins, SYCP1 (green) and SYCP3 (green in PLK1 and INCENP columns) in surface spread spermatocytes progressing from late prophase to late diplotene. Known kinetochore components AURORA B, BUB1, phosphorylated histone H3, CDC20, PLK1, and INCENP are shown in red. CREST is shown in grey. Scale bar represents 5 µm and applies to all panels. (PPTX) [file pgen.1002701.s005.pptx]

## Slide 1
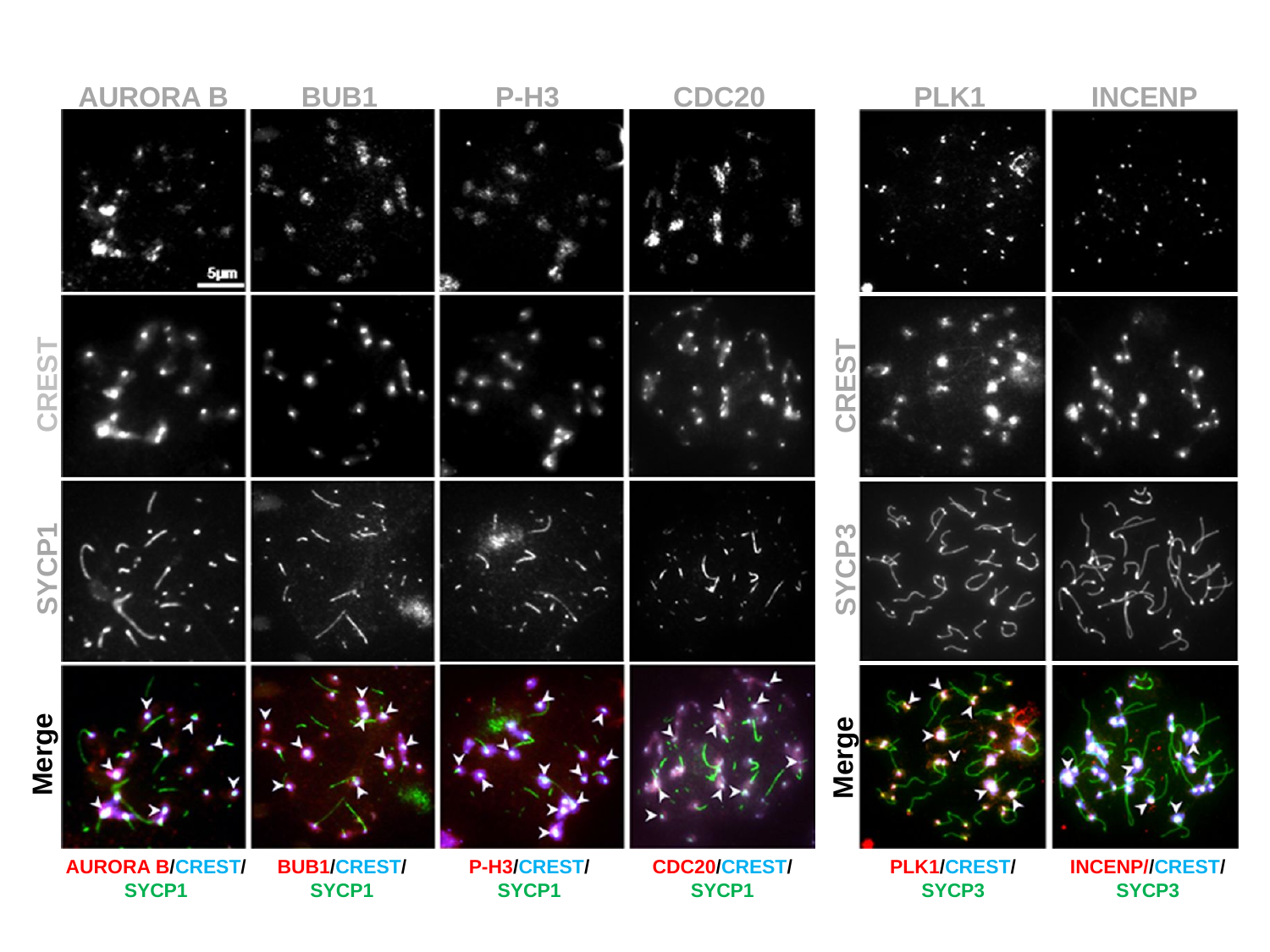

AURORA B
BUB1
P-H3
CDC20
PLK1
INCENP
CREST
CREST
SYCP1
SYCP3
Merge
Merge
AURORA B/CREST/
SYCP1
BUB1/CREST/
SYCP1
P-H3/CREST/
SYCP1
CDC20/CREST/
SYCP1
PLK1/CREST/
SYCP3
INCENP//CREST/
SYCP3
