# Supplementary material for: Synaptonemal Complex Components Persist at Centromeres and Are Required for Homologous Centromere Pairing in Mouse Spermatocytes
Source: PLoS Genet. 2012 Jun 28;8(6):e1002701. doi: 10.1371/journal.pgen.1002701 (PMC3386160; doi:10.1371/journal.pgen.1002701)
Supplement: Text S1 — Protocol for DNA FISH on spermatocyte squash preparations. (DOCX) [file pgen.1002701.s006.docx]

**Supplemental Methods**

**DNA FISH on spermatocyte squash preparations**

Fluorescent in situ hybridization was carried out according to manufacturer’s instructions with a chromosome VIII-specific point probe (ID Lab Inc.).
